# Probiotics Used for Postoperative Infections in Patients Undergoing Colorectal Cancer Surgery

**DOI:** 10.1155/2020/5734718

**Published:** 2020-01-31

**Authors:** Chongxiang Chen, Tianmeng Wen, Qingyu Zhao

**Affiliations:** ^1^Department of Intensive Care Unit, Sun Yat-sen University Cancer Center, State Key Laboratory of Oncology in South China, Collaborative Innovation Center for Cancer Medicine, Guangzhou 510060, China; ^2^Guangzhou Institute of Respiratory Diseases, State Key Laboratory of Respiratory Disease, the First Affiliated Hospital of Guangzhou Medical University, Guangzhou 510120, China; ^3^School of Public Health, Sun Yat-sen University, Guangzhou, Guangdong Province, China

## Abstract

**Objective:**

The objective of this study was to conduct a systematic review and meta-analysis about probiotics to improve postoperative infections in patients undergoing colorectal cancer surgery.

**Methods:**

The PubMed and the Web of Science were used to search for appropriate randomized clinical trials (RCTs) comparing probiotics with placebo for the patients undergoing colorectal cancer surgery. The RevMan 5.3 was performed for meta-analysis to evaluate the postoperative infection, including the total infection, surgical site infection, central line infection, pneumonia, urinary tract infection, septicemia, and postoperative leakage.

**Results:**

Our meta-analysis included 6 studies involving a total of 803 patients. For the incidence of total postoperative infection (odd ratios (OR) 0.31, 95% confidence interval (CI) 0.15–0.64, *I*^2^ = 0%), surgical site infection (OR 0.62, 95% CI 0.39–0.99, *I*^2^ = 0%), surgical site infection (OR 0.62, 95% CI 0.39–0.99, *I*^2^ = 0%), surgical site infection (OR 0.62, 95% CI 0.39–0.99, *I*^2^ = 0%), surgical site infection (OR 0.62, 95% CI 0.39–0.99, *I*^2^ = 0%), surgical site infection (OR 0.62, 95% CI 0.39–0.99, *I*^2^ = 0%), surgical site infection (OR 0.62, 95% CI 0.39–0.99, *I*^2^ = 0%), surgical site infection (OR 0.62, 95% CI 0.39–0.99,

**Conclusions:**

Probiotics is beneficial to prevent postoperative infections (including total postoperative infection, surgical site infection, pneumonia, urinary tract infection, and septicemia) in patients with colorectal cancer.

## 1. Introduction

The postoperative complications of colorectal cancer surgery result in increased ventilation days, hospital stay days, mortality, and cost. Postoperative infection is a major factor affecting the morbidity of the patients. Bacterial translocation is defined as transmitting the bacteria from the gastrointestinal tract to normally sterile tissues. A large number of studies have shown that the bacterial translocation plays a significant role in increasing the incidence of postoperative infections [1, 2].

The probiotics therapy, which was introduced by Lilly and Stillwell [3], could lead to positive clinical and laboratorial outcomes for patients undergoing gastrointestinal surgery. Probiotics are live microorganisms and it is known that probiotics benefit to the host as they can stabilize the intestinal microbiological environment. Nowadays, probiotics have been proved to treat several diseases, such as chronic inflammatory bowel disease [4], hepatic encephalopathy [5], and atopic disease [6]. Horvat et al. [7] showed us his interesting finding that preoperative administration of prebiotics in elective colorectal surgery had the same protective effect in preventing a postoperative inflammatory response as mechanical bowel cleaning.

Probiotics study is very important in recent year, there is a recent paper discussing about the importance of probiotics in the prevention and treatment of colorectal cancer. So we want to conduct a meta-analysis to integrate all this interesting studies to guide clinical practice, as meta-analysis has the higher quality than common RCTs if we only include high quality RCTs. We try to explore the incidence of post-operative infections, including the incidence of the total infection and subgroup infection, such as surgical site infection, central line infection, pneumonia, urinary tract infection, septicemia and postoperative leakage.

## 2. Methods

### 2.1. Search Strategy

**Figure 1:**
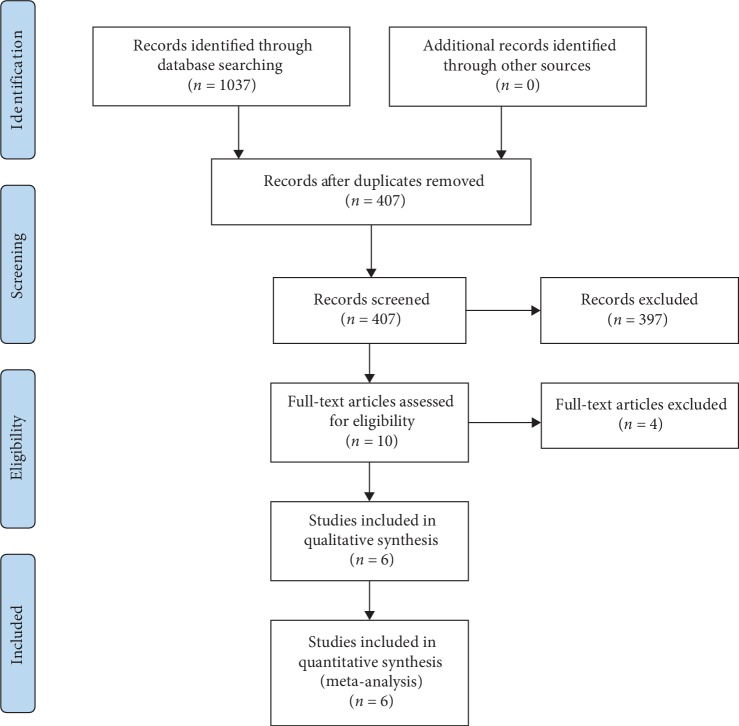
Flow diagram of choosing the appropriated articles.

Two investigators independently searched the articles in the databases (PubMed, the Web of Science). The reference lists of eligible studies and relevant papers were also manually searched and reviewed. Searching terms included “probiotics”, “colorectal cancer”, and “surgery”. Searching terminal date was 2019/1/10. Firstly, we found 407 articles after duplications excluded, and then 307 of them were excluded by reading the title and abstract. Finally, 6 articles were left after reading the whole articles [8–13] (Figure 1).

### 2.2. Inclusion and Exclusion

Inclusions contain: (1) randomized controlled study comparing probiotics with placebo, (2) outcome: various kinds of infections, (3) only be published in English.

Exclusions contain: (1) review, retrospective research, case report, (2) insufficient data in the articles.

### 2.3. Data Elected

**Figure 2:**
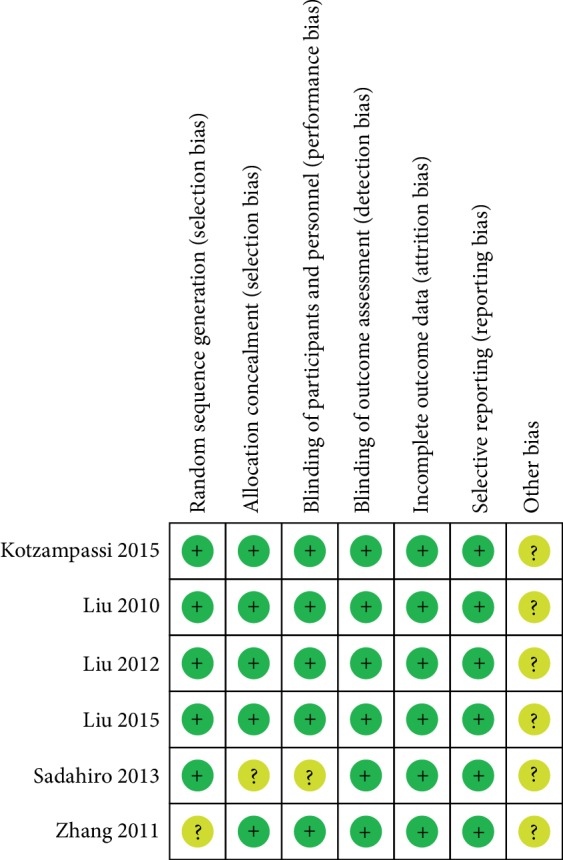
Risk of bias summary.

**Figure 3:**
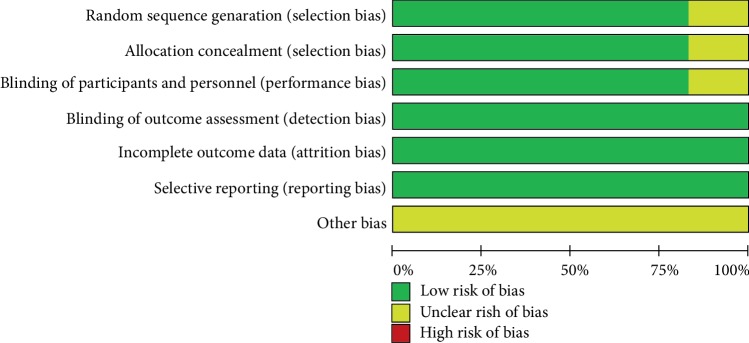
Risk of bias graph.

Two authors (Chongxiang Chen, Tianmeng Wen) independently reviewed the identified abstracts and selected articles to full review. The third reviewer addressed the discrepancies (Qingyu Zhao). For each selected publication, the following baseline and study characteristics were extracted: first author, publication year, country, participant characteristics, patient age, dosage form of probiotics groups, experimental durations, and the baseline characteristics of these studies were concluded (Table 1). The risk of bias of the included studies is shown in Figures 2 and 3. Efficacy outcome measures were the total infection, surgical site infection, central line infection, pneumonia, urinary tract infection, septicemia, and postoperative leakage.

### 2.4. Risk of Bias Assessment

The risk of bias of trials included in this meta-analysis was assessed according to the recommendations of the Cochrane Handbook of Systematic Reviews of Interventions, in the following domains: selection bias (random sequence generation and allocation concealment), performance bias (blinding of participants and personnel), detection bias (blinding of outcome assessment), attrition bias (incomplete outcome data), and reporting bias (selective outcome reporting) (http://handbook.cochrane.org). Jadad scale was used to calculate the quality of every enrolled study.

### 2.5. Statistic Analysis

We pooled data and used mean deviation (OR, with 95% confidence interval) for dichotomy outcomes: the total infection, surgical site infection, central line infection, pneumonia, urinary tract infection, septicemia, and postoperative leakage. We would use a fixed-effect model if there were no considerable heterogeneity among the studies. We would use a random-effects model if the *I*^2^ statistic was above 50% and Cochran's Q statistic had a *P* value ≤0.1. Funnel plots were used to screen for potential publication bias. All statistical analyses were carried out with Review Manager 5.3 (The Cochrane Collaboration).

## 3. Results

The studies included in our meta-analysis were all randomized controlled trials, published from 2010 to 2015. The studies were conducted in Greece [11], China [8, 9, 12, 13], and Japan [10]. Table 1 presents the basic characteristics of included trials and demographic data of participants. All trials were one-center studies and the Jadad Scales of all included studies ranged from 3 to 7.

### 3.1. Total Infection

**Figure 4:**
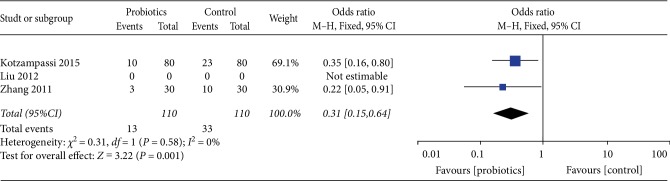
Incidence of total infection.

Comparing probiotics with placebo, our study showed that probiotics could significantly decrease the incidence of postoperative infections. For the total postoperative infection, our study included 3 studies with a total of 220 patients; the results by comparing groups were significantly lower in probiotics group (odd ratios (OR) 0.31, 95% confidence interval (CI) 0.15–0.64). Heterogeneity testing showed that *I*^2^ = 0%, indicating low heterogeneity (Figure 4).

### 3.2. Surgical Site Infection

**Figure 5:**
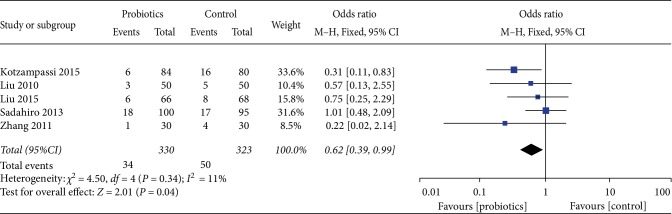
Incidence of postoperative surgical site infection.

For the incidence of surgical site infection, our study included 6 studies involving a total of 653 patients, and the result demonstrated that probiotics was significantly lower than placebo (OR 0.62, 95%CI 0.39–0.99, *I*^2^ = 11%). Heterogeneity testing showed that *I*^2^ = 11%, indicating low heterogeneity. (Figure 5).

### 3.3. Central Line Infection

**Figure 6:**
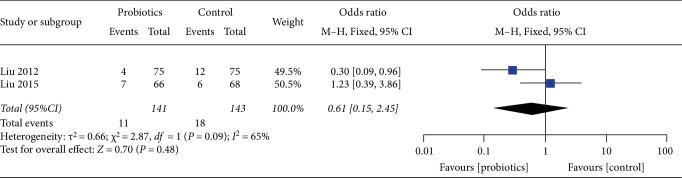
Incidence of postoperative central line infection.

For the results of the incidence of central line infection, our study enrolled 2 studies, including a total of 284 patients, central line infection (OR 0.61, 95%CI 0.15–2.45, *I*^2^ = 65%) reflected no significant difference in two groups. Heterogeneity testing showed that *I*^2^ = 65%, indicating high heterogeneity (Figure 6).

### 3.4. Pneumonia

**Figure 7:**
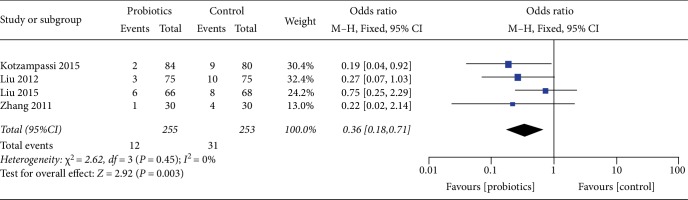
Incidence of postoperative pneumonia.

For the incidence of pneumonia, our study enrolled 4 studies, including a total of 508 patients, and the result showed that probiotics was significantly lower than the placebo (OR 0.36, 95%CI 0.18–0.71, *I*^2^ = 0%). Heterogeneity testing showed that *I*^2^ = 0%, indicating low heterogeneity (Figure 7).

### 3.5. Urinary Tract Infection

**Figure 8:**
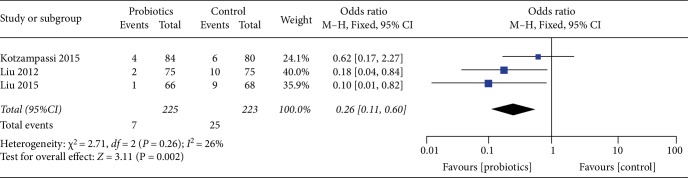
Incidence of postoperative urinary tract infection.

For the result of the incidence of urinary tract infection, our study included 3 studies and a total of 448 patients, and the result reflected significant difference in groups (OR 0.26, 95%CI 0.11–0.60, *I*^2^ = 26%). Heterogeneity testing showed that *I*^2^ = 26%, indicating low heterogeneity (Figure 8).

### 3.6. Septicemia

**Figure 9:**
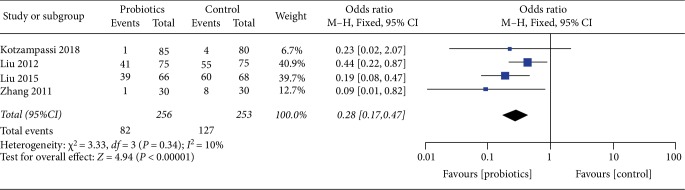
Incidence of postoperative septicemia.

For the result of the incidence of septicemia, our study enrolled 4 studies, including a total of 509 patients, and the result showed that probiotics was significantly lower than the placebo (OR 0.28, 95%CI 0.17–0.47, *I*^2^ = 10%). Heterogeneity testing showed that *I*^2^ = 10%, indicating low heterogeneity (Figure 9).

### 3.7. Postoperative Leakage

**Figure 10:**
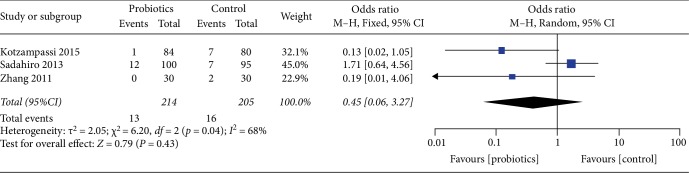
Incidence of postoperative leakage.

For the result of the incidence of postoperative leakage, our study enrolled 3 studies, including a total of 419 patients, and the result did not show that probiotics was significantly lower than the placebo (OR 0.45, 95%CI 0.06–3.27, *I*^2^ = 68%). Heterogeneity testing showed that *I*^2^ = 68%, indicating high heterogeneity (Figure 10).

**Figure 11:**
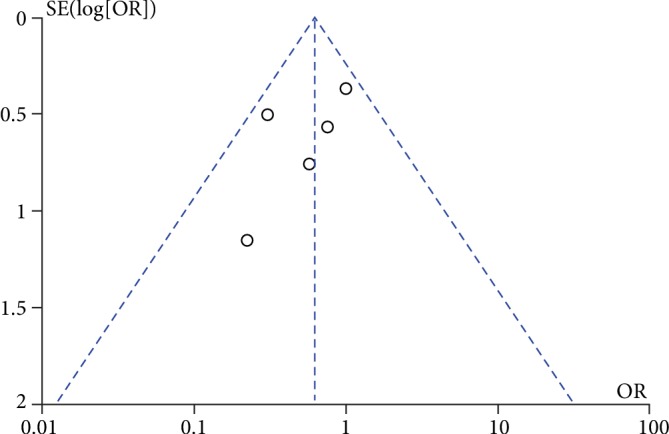
Funnel plot of surgical site infection.

Potential publication bias of probiotics used for surgical site infection was performed and shown as funnel plot (Figure 11).

## 4. Discussion

Several RCTs showed that the use of probiotics in patients with abdominal surgery was a promising approach to the prevention of postoperative infectious complications and well tolerated by patients with minor side effects [14]. However, in abdominal surgery, some investigators reported that there was no evidence supporting any benefit of a preoperative use of probiotics in patients with critical illnesses and undergoing elective abdominal surgery with increased risk of mortality [15, 16], and that in some cases, there was even an increased risk of mortality.

In our meta-analyses, the results showed that probiotics could effectively decrease the rate of postoperative infections, such as pneumonia, surgical site infection, and urinary tract infection.

Not only the incidence of infections but also the quality of life is improved in these studies, which shortens the duration of postoperative hospital stay and the antibiotics administration period. Furthermore, probiotics are considered to generate antitumor agents, which have chemo-preventive effects against colorectal cancer [17]. In addition, probiotics can improve immune function [18].

It is shown that probiotics protect epithelial barrier function. The outcomes probably result from the balance of the enteral bacteria environment. The use of probiotics after surgery markedly improved intestinal microbial populations and significantly decreased the incidence of further infectious complications. The mechanism of the action of probiotics may be related with either the earlier bowel movement preventing bacterial translocation from the gut or the modulation of the innate immune responses.

Gastrointestinal microbiota may be modulated by probiotics. Our study demonstrated that the use of probiotics improved the capacity of the gut ecosystem to survive from surgically induced injury, resulting in fewer postoperative infections. Thus, we concluded that maintaining gut microbiota balance and diversity is important for enhancing host defenses, especially during the recovery from major surgery.

The drawbacks of our study are described as follows: Firstly, we only searched the studies written by English, and the total subjects included in our study were less than the study conducted by Ouyang et al. [19]. Moreover, the combination with prebiotics was not taken into account. Furthermore, the probiotics strain, dose and dosage form in collected studies were not consistent, and the probiotics treatment was combined with other pretreatment modes in some studies, which probably induced certain confusing factors.

However, our study calculated the results of the exactly total subgroups of infection, including the pneumonia, septicemia, central line infection, surgical site infection, postoperative leakage, and urinary tract infection. So our study contains more comprehensive viewpoints of the benefit of probiotics used in colorectal cancer patients undergoing surgery.

## 5. Conclusion

All in all, probiotics is beneficial to prevent postoperative infections (including total postoperative infection, surgical site infection, pneumonia, urinary tract infection, and septicemia) in patients with colorectal cancer. We recommend perioperative oral intake of probiotics as the treatment in patients needing gastrointestinal surgery.

## Figures and Tables

**Table 1:** Baseline characteristics of these studies.

Study	Type	Jadad scale (randomization + concealment of allocation+double blinding + withdrawals and dropouts)	Time (published)	Country	Participant	Total number (probiotics vs. placebo)	Age (probiotics vs. placebo)	Probiotics	Duration
Kotzampassi et al.	RCT	1 + 1 + 2 + 1 = 5	2015	Greece	One center	164; 84/80	Total: ≥18 years; 65.9 ± 11.5 vs. 66.4 ± 11.9	One capsule (*Lactobacillus acidophilus* LA 5, *Lactobacillus plantarum*, *Bifidobacterium lactis* BB 12 *Saccharomyces boulardii*) twice a day	The day of operation and for the next 14 days
Liu et al.	RCT	2 + 1 + 2 + 1 = 6	2012	China (Taiwan)	One RCC	150; 75/75	Total: 25–75 years; 66.06 ± 11.02 vs. 62.28 ± 12.41	Encapsulated bacteria (*Lactobacillus plantarum*; *Lactobacillus acidophilus*11; *Bifidobacterium longum* 88) patients in probiotics group received 2 g/d, at a total daily dose of 2.6 × 10^14^ CFU	6 days preoperatively and 10 days postoperatively
Liu et al.	RCT	2 + 2 + 2 + 1 = 7	2015	China	One ICU	134; 66/68	Total: 25–75 years; 66.62 ± 18.18 vs. 60.16 ± 16.20	Encapsulated probiotics (*Lactobacillus plantarum*; *Lactobacillus acidophilus*11; *Bifidobacterium longum* 88); patients in probiotics group received 2 g/d, at a total daily dose of 2.6 × 10^14^ CFU	Intervention period lasted 16 days, 6 days preoperatively and 10 days postoperatively; Detailed records were recorded for up to 30 days after surgery
Sadahiro et al.	RCT	1 + 1 + 0 + 1 = 3	2013	Japan	One ICU	195; 100/95	Total: 20-80 years; 67 ± 9 vs. 66 ± 12	Three Bifidobacteria tablets orally after each meal three times daily	For 7 days before the operation and from postoperative day 5 for 10 days
Zhang et al.	RCT	1 + 1 + 2 + 1 = 5	2011	China	One center	60; 30/30	Total: None 61.5 (46–82) vs. 67.5 (45–87)	3 oral bifid triple viable capsules (*Enterococcus faecalis*; *Lactobacillus acidophilus*; *Bifidobacterium longum*), 3 times a day	For 3 days (days −5 to −3) before surgery
Liu et al.	RCT	2 + 2 + 2 + 1 = 7	2010	China	One center	100; 50/50	Total: 25–75 years; 65.3 ± 11 vs. 65.7 ± 9.9	Encapsulated bacteria (contain: *Lactobacillus plantarum*; *Lactobacillus acidophilus*; *Bifidobacterium longum*); patients in placebo group received probiotics 2 g/d, total daily dose of 2.6 × 10^14^ CFU	6 days preoperatively and 10 days postoperatively

RCT: randomized controlled trial; ICU: intensive care unit; RCC: respiratory care center.

## Data Availability

The datasets used and/or analyzed in the current study are available from the corresponding author upon request.
